# *De Novo* Design of the ArsR Regulated P_*ars*_ Promoter Enables a Highly Sensitive Whole-Cell Biosensor for Arsenic Contamination

**DOI:** 10.1021/acs.analchem.2c00055

**Published:** 2022-05-10

**Authors:** Sheng-Yan Chen, Yan Zhang, Renjie Li, Baojun Wang, Bang-Ce Ye

**Affiliations:** † School of Chemistry and Chemical Engineering, Shihezi University, Shihezi 832003, China; ‡ College of Chemical and Biological Engineering & ZJU-Hangzhou Global Scientific and Technological Innovation Center, Zhejiang University, Hangzhou 311200, China; § Research Center of Biological Computation, Zhejiang Laboratory, Hangzhou 311100, China; ∥ Centre for Synthetic and Systems Biology, School of Biological Sciences, University of Edinburgh, Edinburgh EH9 3FF, United Kingdom; ⊥ Institute of Engineering Biology and Health, Collaborative Innovation Center of Yangtze River Delta Region Green Pharmaceuticals, College of Pharmaceutical Sciences, Zhejiang University of Technology, Hangzhou 310014, Zhejiang, China; # Lab of Biosystem and Microanalysis, State Key Laboratory of Bioreactor Engineering, East China University of Science and Technology, Shanghai 200237, China

## Abstract

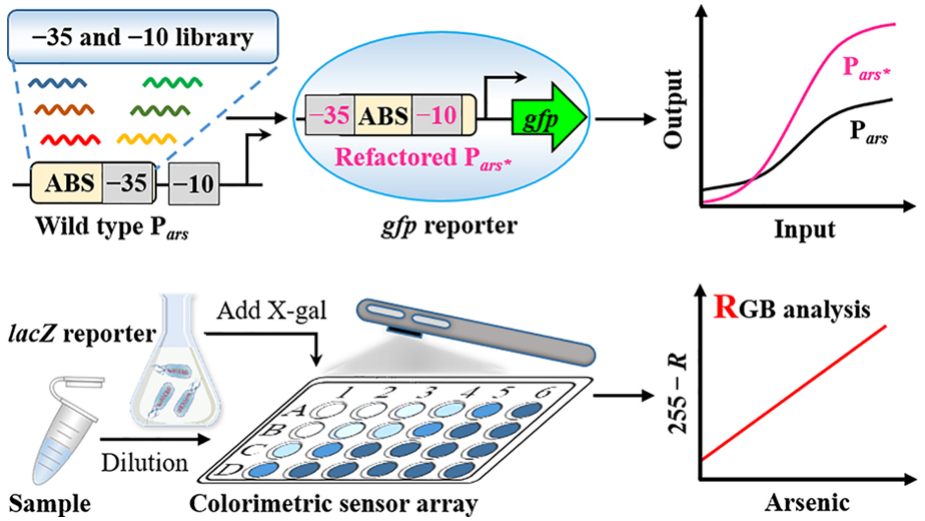

Whole-cell biosensors for arsenic contamination are typically designed based on natural bacterial sensing systems, which are often limited by their poor performance for precisely tuning the genetic response to environmental stimuli. Promoter design remains one of the most important approaches to address such issues. Here, we use the arsenic-responsive ArsR-P_*ars*_ regulation system from *Escherichia coli* MG1655 as the sensing element and coupled *gfp* or *lacZ* as the reporter gene to construct the genetic circuit for characterizing the refactored promoters. We first analyzed the ArsR binding site and a library of RNA polymerase binding sites to mine potential promoter sequences. A set of tightly regulated P_*ars*_ promoters by ArsR was designed by placing the ArsR binding sites into the promoter’s core region, and a novel promoter with maximal repression efficiency and optimal fold change was obtained. The fluorescence sensor P_*lacV*_-P_*arsOC2*_ constructed with the optimized P_*arsOC2*_ promoter showed a fold change of up to 63.80-fold (with green fluorescence visible to the naked eye) at 9.38 ppb arsenic, and the limit of detection was as low as 0.24 ppb. Further, the optimized colorimetric sensor P_*lacV*_-P_*arsOC2*_-*lacZ* with a linear response between 0 and 5 ppb was used to perform colorimetric reactions in 24-well plates combined with a smartphone application for the quantification of the arsenic level in groundwater. This study offers a new approach to improve the performance of bacterial sensing promoters and will facilitate the on-site application of arsenic whole-cell biosensors.

Arsenic contamination of the atmosphere, water, and soil has become a worldwide health issue.^1,2^ It can enter the human body through inhalation, drinking, and even eating, and prolonged exposure to high levels of arsenic can cause serious damage, such as visible skin lesions, peripheral neuropathy, cardiovascular disease, diabetes, and renal system effects.^3−5^ Consequently, the International Agency for Research on Cancer (IARC) classifies arsenic compounds as Group I carcinogens. To assess arsenic pollution and forestall further arsenic exposure, there is an urgent need to develop a rapid and reliable method to determine arsenic.

At present, whole-cell biosensors have attracted the attention of scientists because they are self-renewable and tolerant to harsh environments.^6−8^ Compared with traditional high-end analytical techniques, bacterial biosensors are cost-effective, easy to integrate, portable, and easily applied for high-throughput testing.^9,10^ The development of sensitive whole-cell sensors requires sophisticated sensing and signal transduction elements. Nevertheless, whole-cell sensors based on natural sensing systems have certain shortcomings, such as high leakage, low induced fold change, and poor sensitivity.^11−13^ Recent advances in synthetic biology have provided many methods for improving the regulation of the genetic response to achieve highly sensitive sensors, such as genetic circuit configurations,^14,15^ transcriptional promoter engineering,^16,17^ translational efficiency tuning,^18−21^ posttranslational protein degradation control,^22−24^ and output signal amplifiers.^22,25^ Promoters are the first gate for target gene expression and fundamental elements of the genetic circuit, and selected promoters with excellent performance remain one of the essential considerations for whole-cell biosensor design. The classical prokaryotic inducible promoter (e.g., P_*ars*_, P_*lac*_, and P_*tet*_) comprises two core elements: the RNA polymerase binding sites (−10 and −35 sites) that determine promoter activity and the transcription factor binding sites (TFBSs) that control gene expression. Therefore, promoter engineering usually targets these two core elements for investigation.^16,17,26^ Taking the arsenic-regulated P_*ars*_ promoter as an example, previous studies on promoter engineering have focused on directed evolution (high-throughput screening of optimal promoters),^13^ mutating RNA polymerase binding sites (to enhance or attenuate promoter activity),^12^ adding an additional ArsR binding site (ABS) (to reduce leakage expression),^12,22,27,28^ and changing the relative position of the extra ABS downstream of the promoter (to strengthen or weaken repression).^12,22,27^ These methods all require additional ABSs to reduce the leakage expression, although such additions are detrimental to the sensitivity and signal output of the sensor.

Currently, the *de novo* design of promoters based on TFBS is emerging, which is generally done by embedding TFBS of different affinities within a minimal constitutive promoter or by encoding multiple TFBS (e.g., LacO and TetO) into a single promoter.^16,26^ For transcription-factor-regulated prokaryotic σ^70^ promoters, deploying TFBS at different positions in the promoter will alter the dynamic behavior of the genetic circuit. A consensus on obtaining maximum repression efficiency has been developed for promoters controlled by repression transcription factors (such as LacI/P_*lac*_ and TetR/P_*tet*_).^16,29,30^ The repression efficiency is most robust when the position of the TFBS is located between −10 and −35 sites (approx. 17 bp spacer).^16,26,29−31^ However, transcription factors with long footprint sites are difficult to design (such as ABSs up to 33 bp long).^32^ Shortening the length of TFBS will reduce its affinity for transcription factors and then alter the dynamic behavior of the sensor. Consequently, there has been no research on the *de novo* design of tightly regulated P_*ars*_ promoters to date, thus limiting the further development of arsenic whole-cell sensors. Recent studies predict that promoter sequences are diverse, and up to 60% of random sequences require only single-base mutations to be promoters.^33^ Specific sequences without prominent promoter characteristics still have a strong promoter activity.^34,35^ Such work provides theoretical support for the *de novo* design of tightly regulated promoters controlled by transcription factors with long footprint sites.

To address these challenges, we selected the *Escherichia coli* MG1655 *arsRBC* operon as the starting element for the arsenic sensor circuit analysis, design, and optimization, which can sensitively and specifically recognize arsenic. The sensor circuit (Figure 1A) has a constitutive promoter P_*J109*_ that drives constant expression of the arsenic receptor ArsR. ArsR binds to the ABS to repress P_*ars*_ promoter transcription in the absence of arsenic, which dissociates from the ABS in the presence of arsenic and triggers the reporter gene expression. We collected a library of RNA polymerase binding sites (i.e., −10 site sequences and −35 site sequences) and aligned them to natural ABS sequences to explore potential promoter sequences (Figure 1B). Based on the aligned results, we analyzed the potential promoter model and selected the optimal promoter model for the *de novo* design of promoters based on the principle of maximum overlap between the promoter core region and ABS (Figure 1C). After optimization, two promoters (P_*arsOB4*_ and P_*arsOC2*_) with low leakage and high fold changes were selected, and their applicability was confirmed with *gfp* and *lacZ* as reporter genes, respectively. Ultimately, the low-leakage and highly sensitive P_*lacV*_-P_*arsOC2*_-*lacZ* sensor was used to perform colorimetric reactions in a 24-well transparent plate and combined with a color recognition application of a smartphone to analyze the arsenic content of groundwater samples. This study provided a promising approach for the design of tight regulation promoters and a platform for the rapid detection of arsenic.

**Figure 1:**
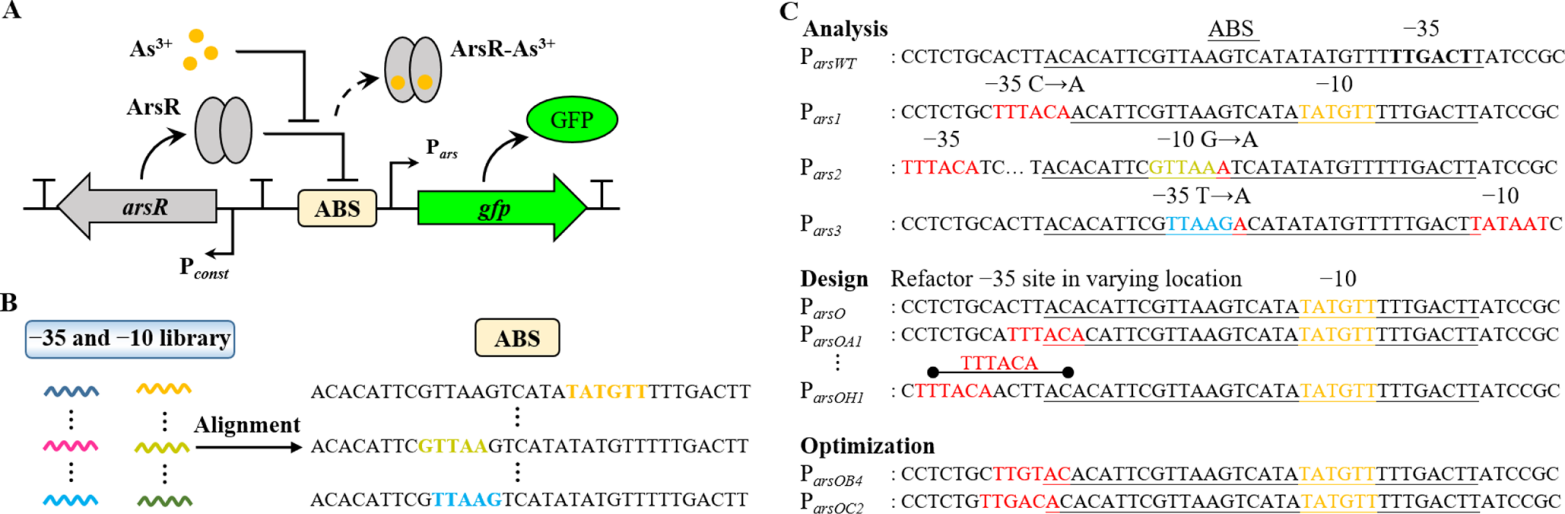
Schematic showing the *de novo* design of the arsenic-responsive tightly regulated P_*ars*_ promoter. (A) Genetic circuit configurations of an arsenic-responsive sensor module coupled to a *gfp* reporter. The dashed arrow indicates the dissociation of ArsR-As^*3+*^ from ABS; the three T-shape structures indicate the terminators. (B) Alignment of a library containing −10 and −35 site sequences with ABS sequences; potential −10 and −35 sites are shown in different colors. (C) Analysis, design, and optimization of tightly regulated P_*ars*_ promoters. The −35 site sequence of the P_*arsWT*_ promoter is shown in bold. Sequences of ABS are underlined. The potential −10 or −35 sites are shown as yellowish (P_*ars1*_), greenish (P_*ars2*_), and blueish (P_*ars3*_) in the three refactored promoters, respectively. The refactored −35 and −10 sites and mutated sequences in ABS are marked in red. The straight lines with dots at both ends indicate that the refactored −35 sites (TTTACA) are sequentially located at six different positions.

## Materials and Methods

### Strains, Growth Conditions, and Reagents

Plasmid construction and biosensor characterization were performed in the *E. coli* DH5α strain. Essential primers and detailed sequences of target genes used in this study are summarized in Tables S1 and S2. Bacteria were cultured in a lysogeny broth (LB) medium supplemented with 30 μg/mL tetracycline antibiotics. Solid media were prepared by supplementation with 15 g/L agar. For bacterial culture, the engineered strains were inoculated from a single colony on freshly streaked plates to 5 mL of LB in sterile 15 mL universal tubes and were incubated overnight at 37 °C with shaking (200 rpm). Unless otherwise noted, data were collected 6 h after the addition of the inducer in all characterization tests. 5-Bromo-4-chloro-3-indolyl β-d-galactopyranoside (X-gal) was purchased from TransGen Biotech. Sodium arsenic (NaAsO_2_) and other chemicals used were analytical grade and purchased from Sigma-Aldrich. The different concentrations of NaAsO_2_ used in this study were all converted to the actual arsenic content for graphical purposes (in ppb).

### Plasmid Circuit Construction

Standard molecular manipulations were used to construct the plasmids by using pPROBE-TT^36^ (pPROBE-TT was a gift from Steven Lindow, Addgene plasmid #37822) as a skeleton. The plasmid carries the *gfp*mut3 gene, referred to as *gfp*, derived from *Aequorea victoria*.^37^ Its product is a green fluorescent protein with a long half-life. The *arsR* gene with the constitutive promoter P_*J109*_ and the ribosome binding site (RBS) B0030 was amplified from the J109-ParsD-ABS-2 plasmid^12^ and inserted into the *Hin*dIII and *Sac*I sites of the sensor detection platform pPROBE-TT plasmid using a recombination reaction to generate the p-TT-P_*J109*_-r30*arsR* plasmid (Figure S1). Primer extension polymerase chain reaction (PCR) was used to obtain the wild-type P_*ars*_ promoter and variant promoters with a refactored −35 site at varying locations. The PCR product was cloned into the *Sac*I and *Eco*RI sites of the p-TT-P_*J109*_-r30*arsR* plasmid to generate the p-TT-P_*J109*_-r30*arsR*-P_*arsXX*_ (*XX* means a different name) plasmids (Figure S2), which were named P_*J109*_-P_*arsXX*_. To change the density of the receptor protein ArsR, the P_*lacV*_ promoter was used to drive ArsR expression. The P_*arsXX*_ gene with the mutation was amplified from the p-TT-P_*J109*_-r30*arsR*-P_*arsXX*_ plasmid by PCR and then ligated with *Eco*RI and *Sac*I restriction-enzyme-digested p-TT-lacV*arsR*^12^ to generate p-TT-P*_lacV_-arsR-*P_*arsXX*_ plasmids, which were named P_*lacV*_-P_*arsXX*_. For the construction of the sensor with *lacZ* as the reporter, *lacZ* with the same RBS as *gfp* was PCR-amplified from the wild-type *E. coli* MG1655 genome and then ligated with *Eco*RI and *Hap*I restriction-enzyme-digested P_*lacV*_-P_*arsXX*_ to generate P_*lacV*_-P_*arsXX*_-*lacZ*. The plasmids used in this study are summarized in Table S3. Genewiz Inc. (Suzhou, China) conducted the oligonucleotide primer synthesis and plasmid sequencing.

### Characterization and Data Analysis of the Fluorescent Biosensor

For fluorescent sensor characterization, the overnight cultures were diluted 50-fold into a fresh LB medium. Then the diluted cultures were loaded into 24-well deep-well plates (Canvic, China) and induced with 100 μL of various concentrations of NaAsO_2_ to a final volume of 2 mL per well. After incubation for 6 h at 37 °C, culture samples of 1 mL were centrifuged for 1 min at 13,400*g*, and the supernatant was decanted. The bacterial pellet was suspended in 1 mL of phosphate-buffered saline (1× PBS, with 1 mg/mL kanamycin to stop the synthesis of GFP), and 200 μL of this suspension was transferred into a clear-bottom 96-well black plate (Fluotrac 200; Greiner, Germany) to measure the cell growth and GFP expression. The OD_600_ (absorbance at 600 nm) and fluorescence (480 nm for excitation, 510 nm for emission, sensitivity = 60%) were read by a microplate reader (Synergy H1 multimode plate reader, BioTek). The PBS averaged backgrounds (*n* = 3, OD_600_ and fluorescence) were determined from wells loaded with 1× PBS and were subtracted from the readings of other wells. The fluorescence/OD_600_ (Fluo/OD_600_) for a sample culture was determined after subtracting the averaged (*n* ≥ 3) counterpart of the negative control cultures (GFP-free) at the same time, and the fold change was calculated as follows:
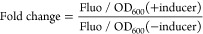


For fold change data, the Fluo/OD_600_ value obtained with inducer NaAsO_2_ was divided by the Fluo/OD_600_ value obtained without inducers. Unless otherwise stated, all fluorescence data were obtained as above. Dose–response curves were fitted using a nonlinear regression model with the Hill slope (log(agonist) vs response – variable slope (four parameters)). All data analyses were performed on GraphPad Prism 7 (GraphPad Software). The limit of detection (LOD) was calculated based on the formula LOD = limit of background (LOB) + 1.645 × SD_(lower concentration)_, where LOB = mean_(blank)_ + 1.645 × SD_(blank)._^22,38,39^ The definition of this equation is based on the fact that the output signal is the concentration of the analyte, so we need to convert the corresponding fluorescence signal to the concentration of arsenic based on the linear equation.

To visualize the expression of GFP, the same induction experiment was performed independently (*n* = 1). Cell pellets held in 2 mL tubes were obtained as described above and photographed under daylight by a Samsung Galaxy S21 cell phone. Meanwhile, the bacterial precipitates were suspended in 1 mL of 1× PBS with 1 mg/mL kanamycin to stop translation, and 200 μL of the bacterial suspension was loaded into a 96-well black plate for fluorescence imaging (λex = 475 nm, λem = 520 nm) using a small-animal imaging system (Night OWL II LB 983 NC100, Berthold, Germany).

### Characterization of the Colorimetric Biosensor

The growth conditions for the engineered sensors are described above. For colorimetric biosensor characterization, the overnight cultures were diluted 50-fold into a fresh LB medium. The chromogenic substrate of 20 mg/L X-gal was supplied to the cell cultures before incubation (unless otherwise indicated, 200 μg/L X-gal was used as the final concentration). Ten microliters of various concentrations of arsenic was added to 96-well clear flat-bottom plates, 190 μL of the culture was added to each well (the induction concentration of arsenic was 0, 1.17, 2.34, 4.69, 9.38, and 18.75 ppb), and the plate was placed in an orbital shaker (200 rpm, 30 °C). Cultures incubated for 6 h were measured for OD_650_ (absorbance at 650 nm) using a BioTek Synergy H1 microplate reader and photographed using a Samsung Galaxy S21 cell phone.

### Colorimetric Analysis of Real Water Samples

A 0.22 μm filter was used to filter the groundwater samples (taken from Mosuwan, Xinjiang) to remove impurities and bacteria. Subsequently, the arsenic content was detected by atomic fluorescence spectrometer (the highest arsenic content in all samples was 20.7 ppb and is referred to as G-Sample 1; all other contents were below 20 ppb). To test the recovery of the sensor for different arsenic levels, G-Sample 2 and G-Sample 3 with arsenic concentrations of 50.7 and 80.7 ppb were prepared by titrating NaAsO_2_ standard samples into G-Sample 1. G-Sample 1, G-Sample 2, and G-Sample 3 were diluted at dilution factors of 1, 2, 3, 4, 5, and 10, and 350 μL of diluted samples and different concentrations of NaAsO_2_ reference samples (with 0, 2, 4, 6, 8, and 10 ppb As) were pre-spiked to clear flat-bottom 24-well plates. Subsequently, 350 μL aliquots of the P_*lacV*_-P_*arsOC2*_-lacZ biosensor cultures (with 400 μg/mL X-gal and 60 μg/mL tetracycline antibiotics) in the logarithmic phase (OD ≈ 0.4) were transferred to each well, and the plate was placed on an orbital shaker (200 rpm, 30 °C). Thus, the final concentrations of X-gal and tetracycline were 200 and 30 μg/mL, respectively, and the arsenic concentrations of the actual and reference samples were further diluted twofold. After 5 h of incubation, colorimetric results of bacterial cultures were acquired according to the method described in the previous section.

### Data Acquisition and Processing

The colorimetric photographs of the 24-well plates were loaded into a mobile app (Color Recognizer) and analyzed for the color intensity of each well. The mobile app can fully analyze the different parameters (such as RGB, CMYK, and LAB) that represented the color intensity. RGB values vary from the range of pure white (255, 255, 255) to pure black (0, 0, 0). As the blue intensity increases, the value of the R channel gradually decreases, so we use 255 minus *R* (255 – *R*) to represent the intensity of blue and establish a linear relationship with the arsenic concentration. The final concentrations of groundwater samples were calculated based on the equation and its dilution factor.

## Results and Discussion

### Refactoring the P_*ars*_ Promoter

To obtain the maximum repression efficiency of ArsR for the P_*ars*_ promoter, the possibility of the maximum overlap between the core region (between the −10 and −35 sites) of the promoter and the ABS was explored. First, the effective length of the ABS was determined by using ABS with variable lengths to reduce the leakage of the arsenic whole-cell biosensors. The results showed that when the ABS sequence was between 24 and 33 bp, the repression efficiency increased with length (Figure S3). Then, we aligned the ABS sequences with the RNA polymerase binding site library (comprising the eighteen −35 sites and thirty-six −10 sites) (Table S4) using the SnapGene software. The alignment results showed that in addition to the wild-type −35 site, there were potential −10 and −35 sites in ABS, which could be generated by one base mutation at most (Figure 1B and Figure S4). Based on these results, we designed three promoter models, P_*ars1*_, P_*ars2*_, and P_*ars3*_ (Figure 1C: Analysis), which provide different options for the *de novo* design of promoters. P_*ars1*_, with the maximal repression efficiency in theory, was selected for further study because its operator site ABS is located in the core region of the promoter and completely overlaps with the −10 site.

**Figure 2:**
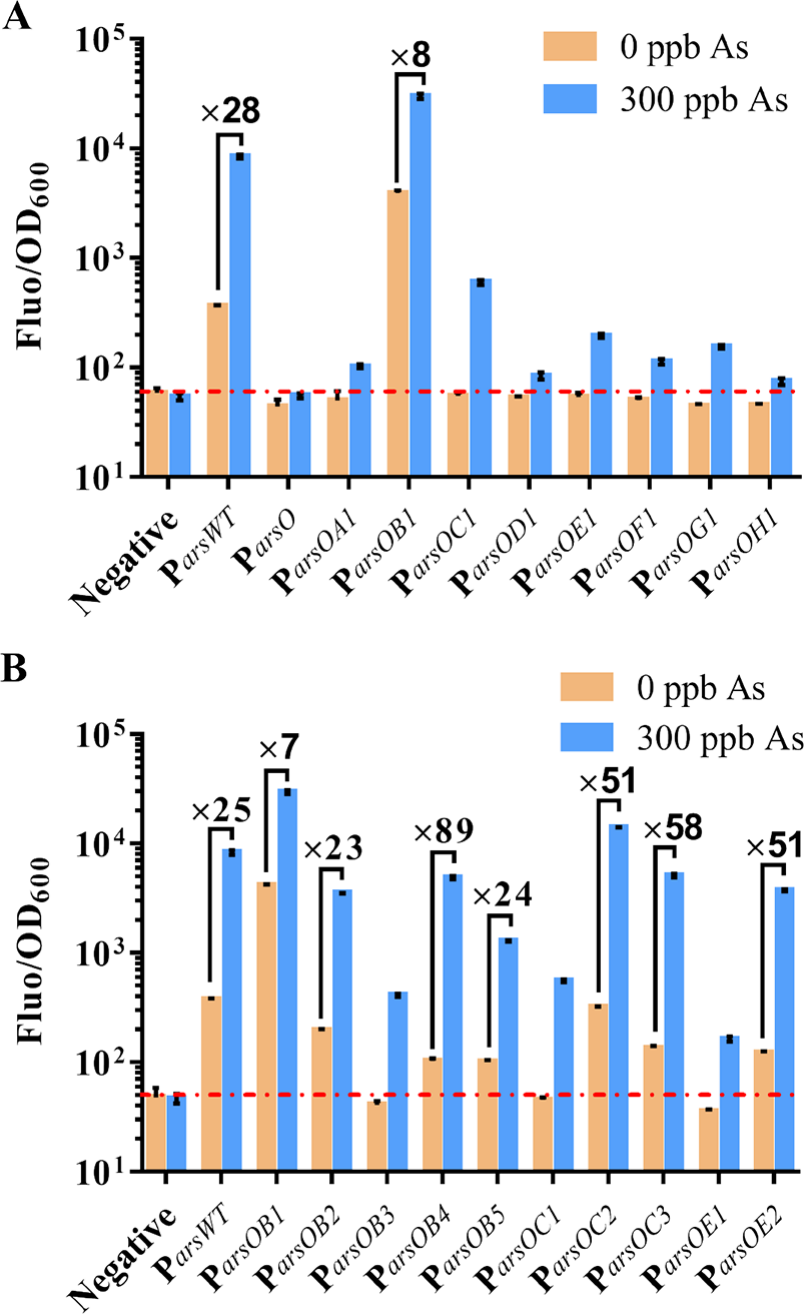
Performance of the refactored promoters (expression of the receptor ArsR is under the control of P_*J109*_) in response to arsenic. (A) The normalized output fluorescence and fold change of the arsenic biosensors comprising the wild-type and refactored promoters. (B) The normalized output fluorescence and fold change of the arsenic biosensors comprising the wild-type and optimized promoters. The red dashed line indicates the output fluorescence level of the negative control. Error bars, standard deviation (*n* = 3).

To test this hypothesis, we refactored a −35 site (TTTACA) by site-directed mutagenesis in different positions upstream of the potential −10 site and eliminated the prototype −10 site of the wild-type promoter (Figure 1C: Design). For P_*arsOA1*_ to P_*arsOH1*_, different capital letters A to H, implying that the refactored −35 sites (TTTACA) are sequentially located at different positions. Although the typical promoter has a spacing of 17 bp between the −10 and −35 regions, we assessed the impact of spacer variations 16 to 23 bp (corresponding to P_*arsOA1*_ to P_*arsOH1*_) on the promoter activity and fold change in the presence of 300 ppb arsenic. Among the nine variants in the first round, the promoter P_*arsOB1*_ with 17 bp spacers was highly active (in the absence and presence of 300 ppb arsenic) with strong fluorescence compared to the unmodified P_*arsWT*_ promoter. Due to the high leakage of P_*arsOB1*_, it exhibited a lower fold change. However, in the presence of 300 ppb arsenic, the signal output of the other promoters was lower than that of the wild-type promoter; in the absence of arsenic, their fluorescence was comparable to that of the negative control (Figure 2A). To avoid generating infinite fold changes, fold changes were not calculated for promoters whose leakage levels were close to the negative control. Nonetheless, as P_*arsOC1*_ and P_*arsOE1*_ possess a longer operator site, these may be more suitable for ArsR with a longer footprint site. We selected P_*arsOB1*_, P_*arsOC1*_, and P_*arsOE1*_ to generate P_*arsOBX*_, P_*arsOCX*_, and P_*arsOEX*_ by refactoring different −35 sites at the same position to further regulate the promoter activity; _*X*_ stands for different numbers, and the same letter with different numbers means that different −35 sites are refactored at the same position (Table S3). In the second round of optimization, P_*arsOB4*_ obtained the highest fold change, and P_*arsOC2*_ obtained a simultaneous improvement in leakage and signal output, thus increasing the fold change to 51× (Figure 2B). These results suggest that improvements in repression efficiency can be achieved by mining the potential promoter sequences to refactor novel promoters. Meanwhile, the performance of modified promoters can continue to be optimized by engineering promoter designs that are not shown here. The current sequences with promoter features were gradually expanded, and a series of new promoters were redesigned. Besides, AI (artificial intelligence)-based promoter redesign and prediction are emerging,^40,41^ thus providing powerful tools for the mining of tightly regulated promoters. Our study gives a new perspective on promoter engineering that could reconstruct artificial promoters with maximal repression efficiency targeted at the specific TFBS.

### Performance of the Fluorescent Biosensor

**Figure 3:**
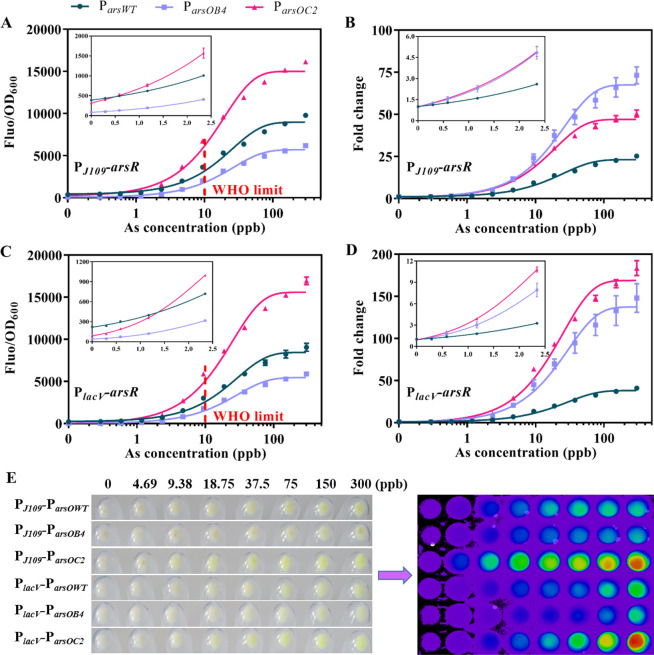
Characterization of the various arsenic-responsive promoters (P_*arsWT*_, circles; P_*arsOB4*_, squares; P_*arsOC2*_, triangle) within a fluorescent whole-cell biosensor. (A–D) Dose–response curves and fold change of different promoters when ArsR expression was driven by P_*J109*_ (A and B) or P_*lacV*_ (C and D). Error bars show the standard deviation (*n* = 3). (E) Images of cell cultures show pellets under daylight (left) or liquids in a fluorescent imaging system (right).

To examine the performance of the refactored promoter variants, we selected P_*J109*_-P_*arsOWT*_, P_*J109*_-P_*arsOB4*_, and P_*J109*_-P_*arsOC2*_ for further analysis. The expression of arsenic receptor ArsR was driven by the P_*J109*_ promoter in these sensors. We investigated the dose–response curves, fold change, and cell phone images after the induction of arsenic at various concentrations. Compared with the wild-type promoter, both P_*arsOB4*_ and P_*arsOC2*_ showed a lower leaky expression in the absence of arsenic, especially P_*arsOB4*_, which was reduced 4.5-fold, whereas P_*arsOC2*_ showed a 2-fold fluorescence increase in the presence of 300 ppb arsenic (Figure 3A). This finding was likely associated with the configuration in which the ABS was located in the core region of the promoter, which reduced the probability of RNA polymerase binding to the promoter in the absence of arsenic and therefore effectively reduced the basal expression. In the presence of 300 ppb of arsenic, the fold change of P_*J109*_-P_*arsWT*_, P_*J109*_-P_*arsOB4*_, and P_*J109*_-P_*arsOC2*_ sensors was 25.27, 73.21, and 50.54, respectively (Figure 3B); moreover, fold changes of 1.12, 1.20, and 1.25 occurred at the lowest induction concentration of 0.29 ppb (Table S5), respectively, indicating that the refactored promoter improved the sensor sensitivity to some extent. Besides, we found that the LOD was somewhat increased for the refactored promoter (Table S5). We speculate that it is due to the increased repression efficiency of ArsR to the refactored promoter. Nonetheless, the difference in LOD between the refactored promoter and the wild-type promoter was less than 0.1 ppb, which is negligible for the sensor development.

Previous studies have shown that the density of arsenic receptor ArsR can modulate the sensitivity and fold change of the sensor.^42^ To show that this improvement in the basal expression, signal output, and sensitivity is robust, we replaced P_*J109*_ with a more potent P_*lacV*_ promoter to express the arsenic receptor ArsR. These results showed that the dose–response curves of the different sensors using the P_*lacV*_ promoter to drive ArsR expression were consistent with those using the P_*J109*_ promoter (Figure 3A,C). Sensors with the P_*lacV*_ promoter possessed a lower leaky expression and hence more remarkable fold changes at the same arsenic level than those using the P_*J109*_ promoter (Figure 3B,D). However, the fold-change of the P_*lacV*_-P_*arsOWT*_ and P_*lacV*_-P_*arsOB4*_ sensors was diminished at low concentrations (Table S5). Interestingly, P_*lacV*_-P_*arsOC2*_ effectively reduced the sensor’s basal expression while maintaining a higher signal output, thus achieving a bidirectional improvement in background noise and signal output. Ultimately, a fold change of 183.52 was produced over the control when induced with 300 ppb arsenic. These fluorescent biosensors were semilog linear (*R*^2^ from 0.9868 to 0.9934) at the range of 2.34 to 150 ppb arsenic (Figure S5). The LOD of the optimal sensor P_*lacV*_-P_*arsOC2*_ was as low as 0.24 ppb, which is 41.67-fold lower than the safety level of 10 ppb for drinking water defined by the World Health Organization.^43^ Therefore, it would fully meet the requirements for real applications.

To visualize the difference in fluorescence of different sensors, we photographed the cell cultures after arsenic induction at different concentrations with and without the fluorescence imaging system (Figure 3E). As shown in Figure 3E (left), the sensor constructed from the P_*arsOC2*_ promoter had green fluorescence visible to the naked eye when the arsenic concentration was higher than 9.38 ppb, while the sensors constructed from the P_*arsOWT*_ and P_*arsOB4*_ promoters required higher concentrations of arsenic to have visible fluorescence. With the increase of arsenic concentration, sensors whose ArsR expression was driven by the P_*lacV*_ promoter exhibited a sensitive color transition (from blue to green, to yellow, and finally to red) than P_*J109*_ (Figure 3E, right). This phenomenon may be due to their lower leaky expression and higher fold changes at the same arsenic concentration.

Since the overexpression of ArsR may affect the specificity of the arsenic sensor,^44^ we performed a specificity assay for P_*J109*_-P_*arsOC2*_ and P_*lacV*_-P_*arsOC2*_ with a wide range of metals that could be potential water contaminants. Remarkably, the expression of the arsenic receptor ArsR using two different promoters showed high specificity for arsenic and no reactivity to the other 10 metal species, even antimony (a homolog of arsenic) (Figure S6A,B). In addition, we tested the performance of the P_*lacV*_-P_*arsOC2*_ sensor at different temperatures. There were a lower signal output and background at 30 °C compared to at 37 °C (Figure S6C), while the fold change was higher at 30 °C (Figure S6D).

### Arsenic Colorimetric Analysis with the β-Galactosidase Biosensor

**Figure 4:**
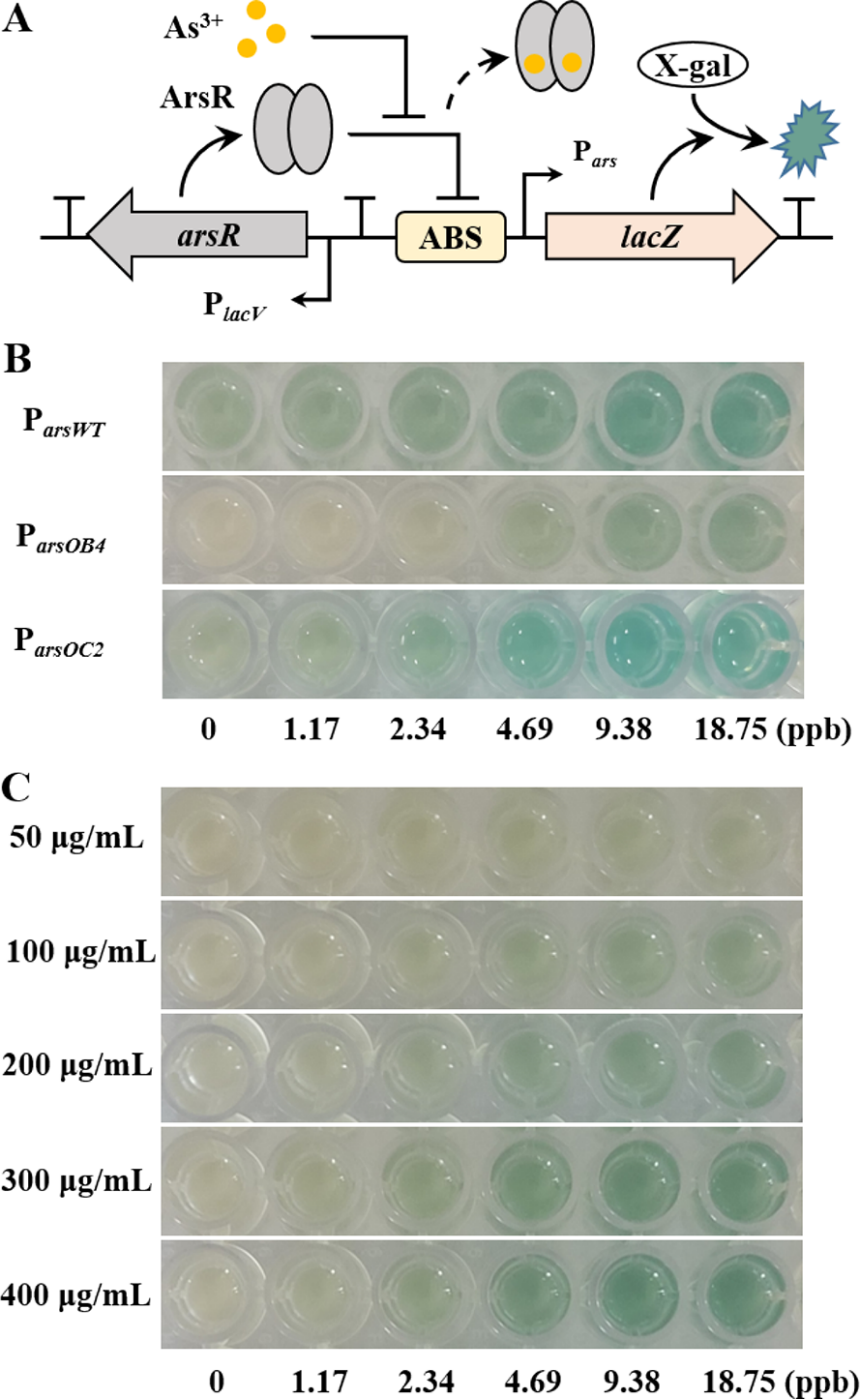
Characterization of various arsenic-responsive promoters within a colorimetric biosensor. (A) Schematic showing the arsenic-responsive sensor module (P_*lacV*_-P_*arsXX*_-lacZ) coupled to a *lacZ* reporter. (B) Images of the colorimetric tests of the three different promoters under various induction levels of arsenic. (C) Images showing the dose responses of the P_*lacV*_-P_*arsOB4*_-*lacZ* sensor to various induction levels of arsenic at different X-gal substrate concentrations.

While it is essential to quantify the level of arsenic, it is more important to quickly diagnose if the arsenic level is over the safe limit. Colorimetric output can be semiquantitatively obtained by observing the color reaction. We replaced *gfp* with *lacZ* as the reporter gene (its product β-galactosidase can cleave the colorless substrate X-gal to blue) (Figure 4A). We first assessed the performance of different promoters at various concentrations of arsenic using 200 μg/mL X-gal as the final concentration of the substrate (Figure 4B). P_*arsWT*_ showed a clear blue color among all promoters tested even in the absence of arsenic, which could easily cause false-positive results in the actual test. P_*arsOB4*_ had an ultralow background with no arsenic, but the blue response was not as pronounced as that of P_*arsOC2*_ when the arsenic concentration increases. We observed that the P_*lacV*_-P_*arsOC2*_-*lacZ* sensor showed a more pronounced blue color at 1.17 ppb than the control; however, when the arsenic content exceeded 4.69 ppb, the blue color intensity would gradually saturate and become indistinguishable. The results of OD_650_ also indicated that the design of a tightly regulated promoter improved the performance of the colorimetric sensor (Figure S7A).

To analyze the effect of X-gal concentration on sensor performance, we tested the P_*lacV*_-P_*arsOB4*_-*lacZ* response to arsenic in different X-gal concentrations. As expected, the blue response of the sensor became more pronounced as the X-gal concentration increased while maintaining a low background (Figure 4C). Thus, the X-gal dosage can effectively adjust the sensor sensitivity to meet different detection requirements. In particular, P_*lacV*_-P_*arsOB4*_-*lacZ* showed a stable OD_650_ for arsenic above 4.69 ppb at 300 and 400 μg/mL X-gal (Figure S7B), which is consistent with the results of the colorimetric reaction. Considering the effect of bacterial growth density on OD_650_, the selection of a suitable color measurement method is important for the quantification of arsenic. For the subsequent experiments, we selected appropriate parameters that allowed the detection of arsenic down to 0–5 ppb while maintaining distinct blue differences. This will allow for a quick assessment of whether arsenic levels exceed the safety level for drinking water defined by the World Health Organization.

### Easy-to-Interpret Colorimetric Array for Arsenic Monitoring

**Figure 5:**
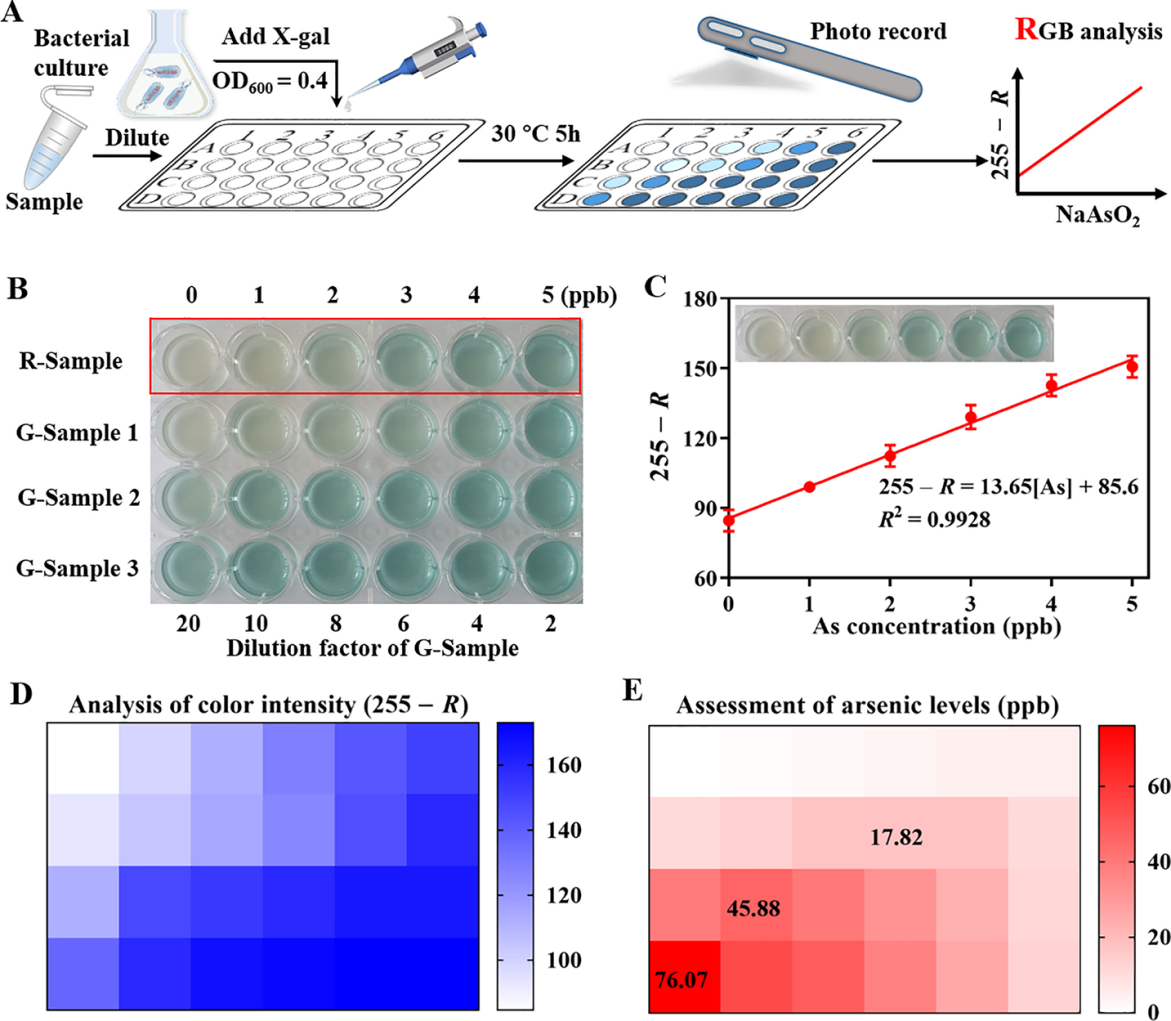
Smartphone-enabled colorimetric assay for arsenic quantification. (A) Schematic showing the detection of arsenic in real samples. (B) Images showing the results of the colorimetric array tests on the reference samples and groundwater samples with different arsenic concentrations. (C) Calibration curve and linear equation for the arsenic concentration and *R* value of reference samples. Error bars, standard deviation (*n* = 3). (D) Color intensity analysis of the colorimetric array with 255 – *R* as the comparison parameter. (E) Arsenic concentration of the actual sample was calculated based on the color intensity (255 – *R*) and the equation 255 – *R* = 13.65(As)/(dilution factor) + 85.6, and the maximum concentration was used as the final result. The heat map shows the mean of three biological replicates.

To enable smartphone portable analysis, we sought to design a colorimetric array based on P_*lacV*_-P_*arsOC2*_-*lacZ* sensors to analyze the actual samples. As shown in Figure 5A, after induction of the sensor by different samples, its color changes were recorded by a smartphone camera, and the different RGB values were read out directly with the help of a smartphone application (Color Recognizer). A functional relationship between the arsenic concentration and color intensity was subsequently established. In principle, the color intensity of the 24-well plates was directly proportional to the concentration of arsenic. However, when the arsenic content exceeded 5 ppb, the blue color gradually saturated and became indistinguishable, which was not conducive to the accurate assessment of arsenic. Therefore, the actual samples needed to be diluted with different dilution factors (2, 4, 6, 8, 10, and 20) first (Figure 5B). Linear relationships between the intensity of blue (255 – *R*) and the arsenic concentration of reference samples (0 to 5 ppb) were established by the P_*lacV*_-P_*arsOC2*_-*lacZ* sensor (Figure 5C). This sensor showed good linearity over the concentration range of 0 to 5 ppb (*R*^2^ = 0.9928), while the LOD was as low as 0.39 ppb, which is 25.64-fold lower than the safety level of 10 ppb for drinking water. For groundwater samples, their color intensity would be within the linear range of the reference samples at the appropriate dilution factor (Figure 5D). The final concentrations of groundwater samples were calculated under different dilution factors based on the equation 255 – *R* = 13.65(As)/(dilution factor) + 85.6. Considering the color saturation phenomenon caused by a high concentration of arsenic, we chose the maximum concentration as the assessment result of arsenic risk (Figure 5E).

As shown in Table S6, the recovery of arsenic measured by the P_*lacV*_-P_*arsOC2*_-*lacZ* biosensor was in the range of 86.08 to 94.26%, which was exceedingly good based on the actual concentration of groundwater. Due to the sensor’s high sensitivity, it could distinguish color differences in final concentrations of arsenic of 0–5 ppb. It is theoretically possible to accurately assess arsenic levels from 0 to 100 ppb (or even higher if the dilution factor is increased) after dilution conversion. Meanwhile, the mobile app is a scalable platform that can be programmed to convert the signal output from RGB to the arsenic concentration, which will facilitate and accelerate the detection and the field application of arsenic.

## Conclusions

In summary, to address the issues of high background and low induction fold of arsenic whole-cell biosensors, we proposed a *de novo* promoter design approach for transcription-factor-regulated promoters with long footprint sites. The design approach is time-saving compared to error-prone PCR and high-throughput screening methods. We mined potential promoters within the ABS and developed improved arsenic whole-cell biosensors with low leakage and high signal output. The fold change of the optimized sensor was increased from the initial 25.27× to 187.52× in the presence of 300 ppb arsenic. Compared to previous studies, we constructed P_*lacV*_-P_*arsOC2*_ and P_*lacV*_-P_*arsOC2*_-*lacZ* sensors that showed a superior detection limit and linear range (Table S7). Excess ArsR may be detrimental to the detection limit and sensitivity of the sensor, while our refactored promoter greatly improves the repression efficiency of ArsR to P_*ars*_ and avoids the use of excess ArsR to control the basal expression. Hence, this approach enables the repression of background noise and increase of fold change upon signal emission without impairing the detection limit and sensitivity. In addition, excess ArsR increases the nonspecificity of the sensor. Therefore, the *de novo* designed promoter provides a new alternative approach for the development of arsenic sensors with a low detection limit, high signal-to-noise ratio, and high specificity. The use of different reporter genes to construct the sensor showed different characteristics, with the *gfp* reporter requiring a higher expression to obtain a visible output signal though having a wider linear range, while the *lacZ* reporter facilitates the development of a device-free detection platform due to the chromogenic response though with a narrower linear range and requiring a lower promoter leakage. Low leakage and high signal output promoters allow for a more flexible selection of reporter genes and are the basis for obtaining highly sensitive sensors. Finally, a highly sensitive colorimetric sensor array was built to accurately assess arsenic contaminant levels from 0 to 100 ppb and had a low detection limit of 0.39 ppb. Although the current sensor requires 5 h of incubation to generate a sufficiently strong colorimetric output for visualization, this is mainly associated with the restricted diffusion and transport of substrates across the cell membrane. This can be optimized to generate a much faster response by cell-free systems or lysing cells.^8,45^ The color difference arising from the colorimetric array assay can be quickly captured by a smartphone without sophisticated equipment, thus facilitating its potential use as an easy-to-integrate and low-cost environmental monitoring tool in the field. This study provides a new approach for designing tightly regulated promoters with an aim for developing highly sensitive whole-cell biosensors. It will facilitate the on-site application of whole-cell biosensors for precise arsenic detection.

## Supporting Information Available

Schematic diagram of the p-TT-P*J109*-r30*arsR* plasmid construct (Figure S1); schematic diagram of the p-TT-P*J109*-r30*arsR*-P*arsXX* (named P*J109*-P*arsXX*) plasmid construct (Figure S2); validation of the effective length of the ArsR binding site (Figure S3); results showing the ABS sequence alignment with the RNA polymerase binding site (Figure S4); different fluorescent sensors showing a linear response to arsenic (Figure S5); sensor specificity and temperature effect on the sensor performance (Figure S6); absorbance of diverse colorimetric sensors at 650 nm monitored across various arsenic concentrations (Figure S7); primers used in this study (Table S1); sequences of the genetic constructs used in this study (Table S2); plasmid constructs used in this study (Table S3); RNAP binding site library containing eighteen −35 sites and thirty-six −10 sites (Table S4); best fits for the characterized responses of the arsenic sensors with diverse promoters (Table S5); accuracy and reliability of the constructed biosensors for real samples (Table S6); and comparison of previously reported arsenic sensors with this study (Table S7) (PDF)
