# Supplementary material for: De Novo Design of the ArsR Regulated Pars Promoter Enables a Highly Sensitive Whole-Cell Biosensor for Arsenic Contamination
Source: Anal Chem. 2022 May 10;94(20):7210–8. doi: 10.1021/acs.analchem.2c00055 (PMC9134189; doi:10.1021/acs.analchem.2c00055)
Supplement: Supplementary file 1 — ac2c00055_si_001.pdf [file ac2c00055_si_001.pdf]

Supporting Information

De novo design of the ArsR regulated P_{ars} promoter enables a highly sensitive whole-cell biosensor for arsenic contamination

Sheng-Yan Chen[a], Yan Zhang[a], Renjie Li[a], Baojun Wang[b, c, d*], and Bang-Ce Ye[a, e, f*]

[a]School of Chemistry and Chemical Engineering, Shihezi University, Shihezi 832003, China.
[b]College of Chemical and Biological Engineering & ZJU-Hangzhou Global Scientific and Technological Innovation Center, Zhejiang University, Hangzhou 311200, China.
[c]Research Center of Biological Computation, Zhejiang Laboratory, Hangzhou 311100, China.
[d]Centre for Synthetic and Systems Biology, School of Biological Sciences, University of Edinburgh, Edinburgh EH9 3FF, United Kingdom.
[e]Institute of Engineering Biology and Health, Collaborative Innovation Center of Yangtze River Delta Region Green Pharmaceuticals, College of Pharmaceutical Sciences, Zhejiang University of Technology, Hangzhou 310014, Zhejiang, China.
[f]Lab of Biosystem and Microanalysis, State Key Laboratory of Bioreactor Engineering, East China University of Science and Technology, Shanghai 200237, China.

*Corresponding author: Bang-Ce Ye, E-mail: bcye@ecust.edu.cn. Baojun Wang, E-mail: baojun.wang@zju.edu.cn.

Table of Contents

Figure S1. Schematic diagram of the p-TT-P_{J109}-r30*arsR* plasmid construct 2

Figure S2. Schematic diagram of the p-TT-P_{J109}-r30*arsR*-P_{arsXX} (named as P_{J109}-P_{arsXX}) plasmid construct 2

Figure S3. Validation of the effective length of the ArsR binding site 3

Figure S4. Results showing ABS sequence alignment with the RNA polymerase binding site 3

Figure S5. Different fluorescent sensors showing linear response to arsenic 3

Figure S6. Sensor specificity and temperature effect on sensor performance 4

Figure S7. Absorbance of diverse colorimetric sensors at 650 nm was monitored across various arsenic concentrations 4

Table S1. Primers used in this study 5

Table S2. Sequences of the genetic constructs used in this study 6

Table S3. Plasmid constructs used in this study 6

Table S4. RNAP binding site library contains eighteen −35 sites and thirty-six −10 sites 10

Table S5. Best fits for the characterized responses of the arsenic sensors with diverse promoters 11

Table S6. Accuracy and reliability of the constructed biosensors for real samples 11

Table S7. Comparison of previously reported arsenic sensors with this study 11

References 12

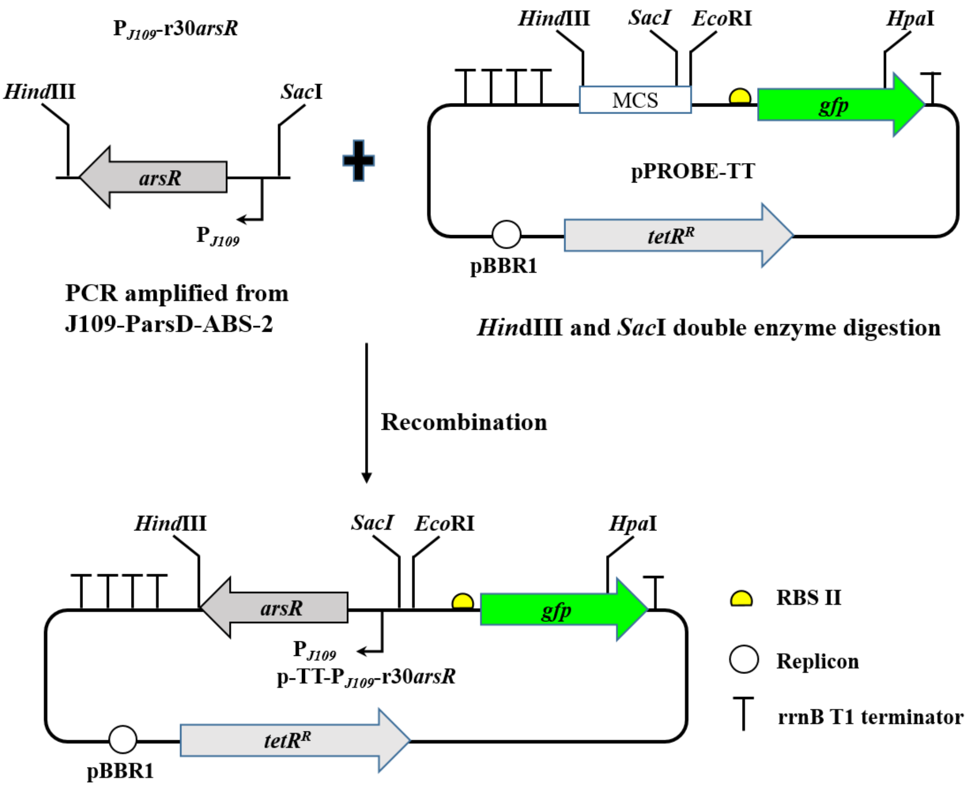

Figure S1. Schematic diagram of the p-TT-P$_{J109}$-r30*arsR* plasmid construct.

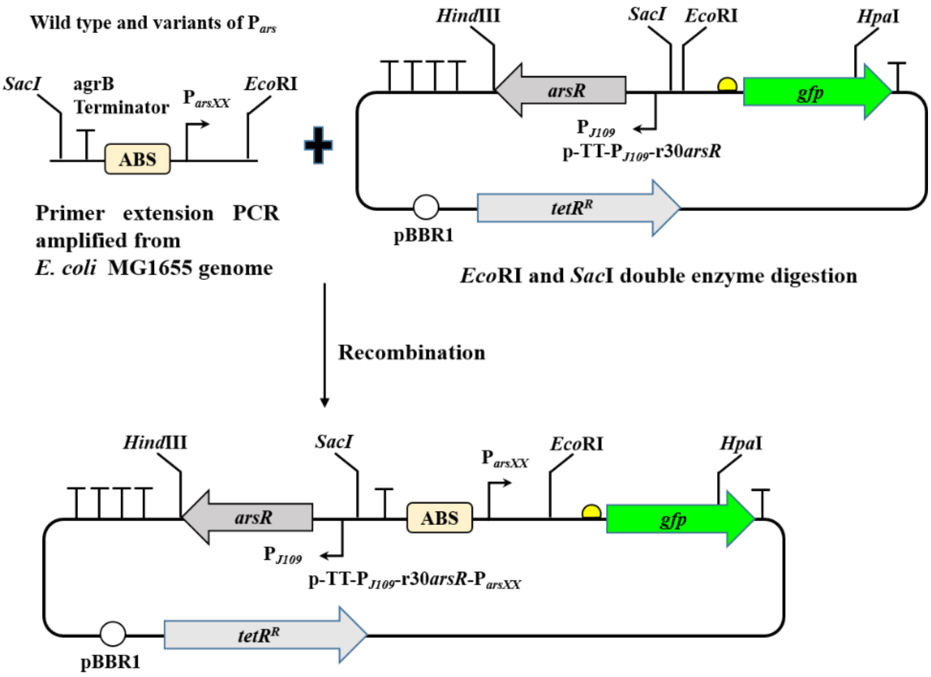

Figure S2. Schematic diagram of the p-TT-P$_{J109}$-r30*arsR*-P$_{arsXX}$ (named as P$_{J109}$-P$_{arsXX}$) plasmid construct.

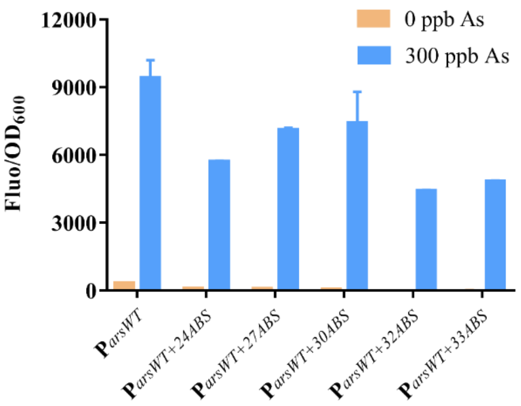

Figure S3. Validation of the effective length of the ArsR binding site.

The ABS of different lengths were added downstream of the wild-type promoter to test the leakage level of the sensor.

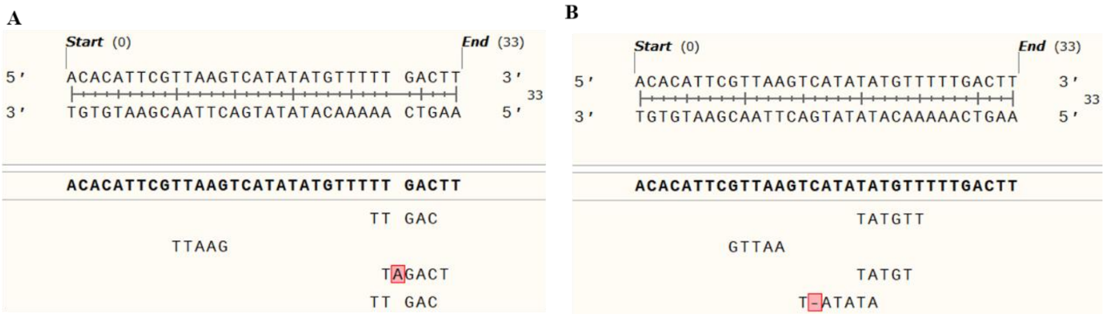

Figure S4. Results showing ABS sequence alignment with the RNA polymerase binding site.

(**A**) Alignment with the −35 sites library allows the ABS sequence to generate new −35 sites.
(**B**) Alignment with the −10 sites library allows the ABS sequence to generate new −10 sites.

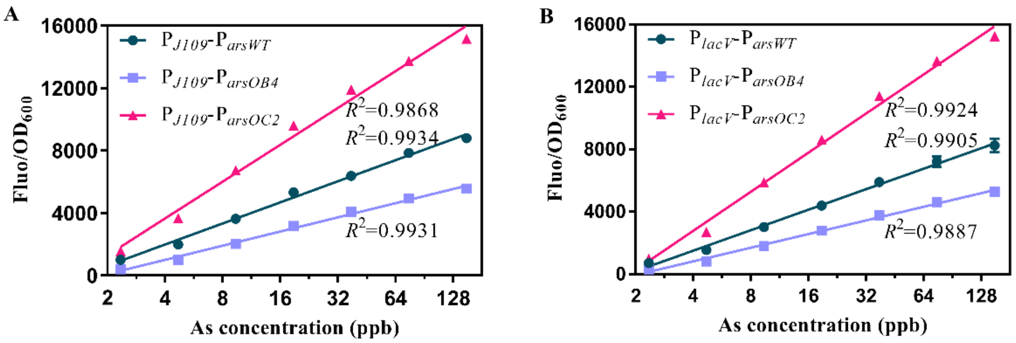

Figure S5. Different fluorescent sensors showing linear response to arsenic.

(**A**) Best-fit parameters for the P_{J109}-P_{arsWT}, P_{J109}-P_{arsOB4}, and P_{J109}-P_{arsOC2} biosensors in the 2.34 to 150 ppb arsenic concentration. (**B**) Best-fit parameters for the P_{lacV}-P_{arsWT}, P_{lacV}-P_{arsOB4}, and P_{lacV}-P_{arsOC2} biosensors in the 2.34 to 150 ppb arsenic concentration. The data from Figure 3.A and C, the error bars are smaller than the data symbols (n = 3).

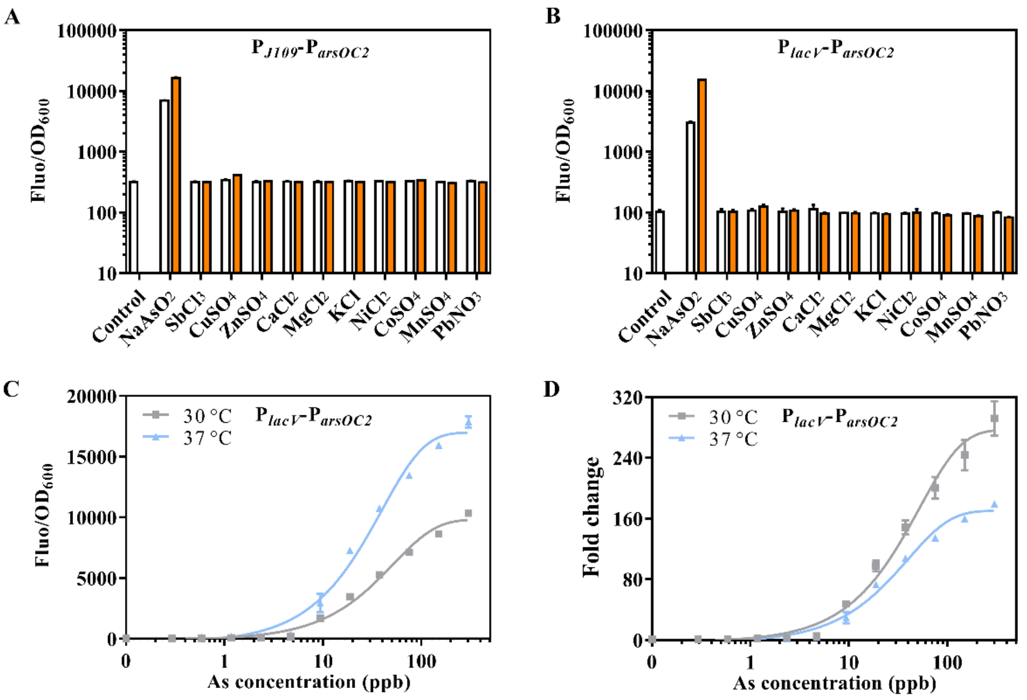

Figure S6. Sensor specificity and temperature effect on sensor performance.

(**A-B**) Characterization of the specificity of the P_{J109}-P_{arsOC2} and P_{lacV}-P_{arsOC2} sensor, for arsenic and antimony, the induced final concentrations were 0.1 µM (7.5 ppb for As) and 1 µM (75 ppb for As); for all other compounds, the induced concentrations were 10 µM and 100 µM. (**C-D**) The dose-response and fold change of the P_{lacV}-P_{arsOC2} sensor to different concentrations of arsenic at 30 °C and 37 °C. Error bars show the standard deviation (n = 3).

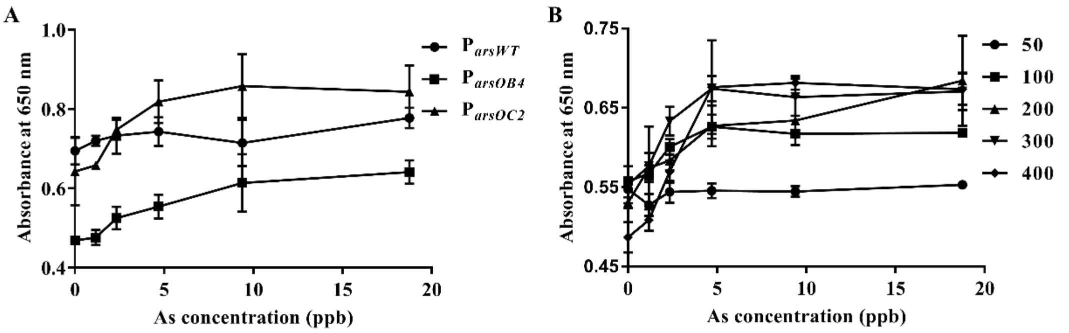

Figure S7. Absorbance of diverse colorimetric sensors at 650 nm was monitored across various arsenic concentrations.

(**A**) OD_{650} of cell cultures with different colorimetric sensors at various concentrations of arsenic (X-gal = 200 µg/mL). (**B**) OD_{650} of the P_{lacV}-P_{arsOB4}-*lacZ* sensor at different X-gal substrate concentrations. Error bars show the standard deviation (n = 3).

Table S1. Primers used in this study

Primer name	Sequence
HR-EcoRI-Pars-R	gttagttagggaataagccgaattcTTGTTGCAGGTAGTGTCTCTCTTCG
arsO-R1	AGGAAGGTAATAGGTGTGAATTTTG
arsO-R3	gggaataagccgaattcTTCGAAGCGGATAAGTCAAAAACATATATGACTTAACGAATGT
arsOA1-R2	AAAACATATATGACTTAACGAATGTGTAAATGCAGAGGAAGGTAATAGGTGTGAATTTTG
arsOB1-R2	AAAACATATATGACTTAACGAATGTTGTAAAGCAGAGGAAGGTAATAGGTGTGAATTTTG
arsOB2-R2	AAAACATATATGACTTAACGAATGTGGTAAAGCAGAGGAAGGTAATAGGTGTGAATTTTG
arsOB3-R2	AAAACATATATGACTTAACGAATGTGTTAAAGCAGAGGAAGGTAATAGGTGTGAATTTTG
arsOB4-R2	AAAACATATATGACTTAACGAATGTGTACAAGCAGAGGAAGGTAATAGGTGTGAATTTTG
arsOB5-R2	AAAACATATATGACTTAACGAATGTGTAAAAGCAGAGGAAGGTAATAGGTGTGAATTTTG
arsOC1-R2	AAAACATATATGACTTAACGAATGTGTGTAAACAGAGGAAGGTAATAGGTGTGAATTTTG
arsOC2-R2	AAAACATATATGACTTAACGAATGTGTGTCAACAGAGGAAGGTAATAGGTGTGAATTTTG
arsOC3-R2	AAAACATATATGACTTAACGAATGTGAGTCAACAGAGGAAGGTAATAGGTGTGAATTTTG
arsOD1-R2	AAAACATATATGACTTAACGAATGTGTTGTAAAAGAGGAAGGTAATAGGTGTGAATTTTG
arsOE1-R2	AAAACATATATGACTTAACGAATGTGTATGTAAAGAGGAAGGTAATAGGTGTGAATTTTG
arsOE2-R2	AAAACATATATGACTTAACGAATGTGTATGTCAAGAGGAAGGTAATAGGTGTGAATTTTG
arsOF1-R2	AAAACATATATGACTTAACGAATGTGTAATGTAAAAGGAAGGTAATAGGTGTGAATTTTG
arsOG1-R2	AAAACATATATGACTTAACGAATGTGTAAGTGTAAAGGAAGGTAATAGGTGTGAATTTTG
arsOH1-R2	AAAACATATATGACTTAACGAATGTGTAAGTTGTAAAGAAGGTAATAGGTGTGAATTTTG
ParsWT24arsO-R	CGAATTCGCGGAAAGTCAAAAACATATATGACTTAATTGTTGCAGGTAGTGTCTCTCTTC
ParsWT27arsO-R	CGAATTCGCGGAAAGTCAAAAACATATATGACTTAACGATTGTTGCAGGTAGTGTCTCTCTTC
ParsWT30arsO-R	CGAATTCGCGGAAAGTCAAAAACATATATGACTTAACGAATGTTGTTGCAGGTAGTGTCTCTCTTC

ParsWT32arsO-R	CGAATTCGCGGAAAGTCAAAAACATATATGACTTAACGAATGTGTTGTTGCAGGTAGTGTCTCTCTTC
ParsWT33arsO-R	CGAATTCGCGGAAAGTCAAAAACATATATGACTTAACGAATGTGTTTGTTGCAGGTAGTGTCTCTCTTC
(pTTsacI)J109-R	gaataagccgaattcgagctcTTTACAGCTAGCTCAGTCCTAGGGAC
(J109)Pars-F	ctaggactgagctagctgtaaagagctcAGCCACTGGCTAATAGTAT
(pTTsacI)PlacV-R	gaataagccgaattcgagctcGCGCCCAATACGCAAACCG
(lacVsacI)Pars-F	ggtttgcgtattgggcgcgagctcAGCCACTGGCTAATAGTATTGAGCTG
(pTTRBS)lacZ-F	CTAACTAAAGATTAACTTTATAAGGAGGAAAAACATATGACCATGATTACGGATTCACTG
(Parswt)pTTRBS-F	tacctgcaacaagaattcGGCTTATTCCCTAACTAACTAAAGATTAACTTTATAAGGAGG
(arsO)pTTRBS-F	atccgcttcgaagaattcGGCTTATTCCCTAACTAACTAAAGATTAACTTTATAAGGAGG
(HRhapI)lacZ-R	ttgtgtctaattttgaagttaacTTATTATTTTTGACACCAGACCAACTG
pPROBE-TT-F	GGAATTGGGGATCGGAAGCTT (sequencing primer)
pPROBE-TT-R	GCATCACCTTCACCCTCTCCAC (sequencing primer)

Note: The DNA sequence (lowercase) indicates the homologous recombination sequence on the plasmid. The underlined sequences are restriction endonuclease sites.

Table S2. Sequences of the genetic constructs used in this study

Gene name	Sequence and characteristic
P_{J109} -RBS30	(*Sac*I)TTTACAGCTAGCTCAGTCCTAGGGACTGTGCTAGCTACTAGAGATTAAAGAGGAGAAATACTAG(*arsR*)<br>RBS30: ATTAAAGAGGAGAAATACTAG
P_{lacV}-RBS$_{arsR}$	(*Sac*I)ACGCAAACCGCCTCTCCCCGCGCGTTGGCCGATTCATTAATGCAGCTGGCACGACAGGTTTCCCGACTGGAAAGCGGGCAGTGAGCGCAACGCAATTAATGTGAGTTAGCTCACTCATTAGGCACCCCAGGC**TTTACG**TTGTGAGCGCTCACAAT**TATAGT**GTGTGGAATCAATCAGGAGCGCAAT (*arsR*)<br>RBS$_{arsR}$: CAATCAGGAGCGCAAT
arsR	(P_{J109} -RBS30 or P_{lacV}-RBS$_{arsR}$)<br>ATGTCATTTCTGTTACCCATCCAATTGTTCAAAATTCTTGCTGATGAAACCCGTCTGGGCATCGTTTTACTGCTCAGCGAACTGGGAGAGTTATGCGTCTGCGATCTCTGCACTGCTCTCGACCAGTCGCAGCCCAAGATCTCCCGCCACCTGGCATTGCTGCGTGAAAGCGGGCTATTGCTGGACCGCAAGCAAGGTAAGTGGGTTCATTACCGCTTATCACCGCATATTCCAGCATGGGCGGCGAAAATTATTGATGAGGCCTGGCGATGTGAACAGGAAAAGGTTCAGGCGATTGTCCGCAACCTGGCTCGACAAAACTGTTCCGGGGACAGTAAGAACATTTGCAGTTAA(*Hin*dIII)

P$_{arsWT}$ promoter	(*Sac*I)AGCCACTGGCTAATAGTATTGAGCTGTTAGATAAGAACTCTCTCACTCCAGCCAG<br>AGCCACCAACTCAGGGCTGGAAAGT**AAAAAACCGACGCAAAGTCGGTTTTTTTACG**<br>**TCCTGA**TTCAGACCTCCTTTCAAATGAATAGCCAACTCAAAATTCACACCTATTACCTT<br>CCTCTGCACTTACACATTCGTTAAGTCATATATGTTT**TTGACT**TATCCGCTTCGAAGAG<br>A**GACACT**ACCTGCAACAA (*Eco*RI)<br>agrB Terminator: **AAAAAACCGACGCAAAGTCGGTTTTTTTACGTCCTGA**[1]<br>ArsR Binding site: ACACATTCGTTAAGTCATATATGTTT**TTGACT**T[2]<br>−35 site: **TTGACT**<br>−10 site: **GACACT**
gfp gene (pPROBE-TT carrying a *gfp*)	(*Eco*RI)ggcttattccctaactaactaaagattaactttataaggaggaaaaacatATGAGTAAAGGAGAAGAACTTTT<br>CACTGGAGTTGTCCCAATTCTTGTTGAATTAGATGGTGATGTTAATGGGCACAAATTTTC<br>TGTCAGTGGAGAGGGTGAAGGTGATGCAACATACGGAAAACTTACCCTTAAATTTATTT<br>GCACTACTGGAAAACTACCTGTTCCATGGCCAACACTTGTCACTACTTTGACTTATGGT<br>GTTCAATGCTTTTCAAGATACCCAGATCATATGAAACGGCATGACTTTTTCAAGAGTGC<br>CATGCCCGAAGGTTATGTACAGGAAAGAACTATATTTTTCAAAGATGACGGGAACTACA<br>AGACACGTGCTGAAGTCAAGTTTGAAGGTGATACCCTTGTTAATAGAATCGAGTTAAAA<br>GGTATTGATTTTAAAGAAGATGGAAACATTCTTGGACACAAATTGGAATACAACTATAA<br>CTCACACAATGTATACATCATGGCAGACAAACAAAAGAATGGAATCAAAGTTAACTTCA<br>AAATTAGACACAACATTGAAGATGGAAGCGTTCAACTAGCAGACCATTATCAACAAAA<br>TACTCCAATTGGCGATGGCCCTGTCCTTTTACCAGACAACCATTACCTGTCCACACAATC<br>TGCCCTTTCGAAAGATCCCAACGAAAAGAGAGACCACATGGTCCTTCTTGAGTTTGTA<br>ACAGCTGCTGGGATTACACATGGCATGGATGAACTATACAAATAG
lacZ gene (Amplified from *E. coli* MG1655 by PCR)	(*Eco*RI)ggcttattccctaactaactaaagattaactttataaggaggaaaaacatATGACCATGATTACGGATTCACTG<br>GCCGTCGTTTTACAACGTCGTGACTGGGAAAACCCTGGCGTTACCCAACTTAATCGCCT<br>TGCAGCACATCCCCCTTTCGCCAGCTGGCGTAATAGCGAAGAGGCCCGCACCGATCGC<br>CCTTCCCAACAGTTGCGCAGCCTGAATGGCGAATGGCGCTTTGCCTGGTTTCCGGCACC<br>AGAAGCGGTGCCGGAAAGCTGGCTGGAGTGCGATCTTCCTGAGGCCGATACTGTCGTC<br>GTCCCCTCAAACTGGCAGATGCACGGTTACGATGCGCCCATCTACACCAACGTGACCTA<br>TCCCATTACGGTCAATCCGCCGTTTGTTCCCACGGAGAATCCGACGGGTTGTTACTCGC<br>TCACATTTAATGTTGATGAAAGCTGGCTACAGGAAGGCCAGACGCGAATTATTTTTGAT<br>GGCGTTAACTCGGCGTTTCATCTGTGGTGCAACGGGCGCTGGGTCGGTTACGGCCAGG<br>ACAGTCGTTTGCCGTCTGAATTTGACCTGAGCGCATTTTTACGCGCCGGAGAAAACCG<br>CCTCGCGGTGATGGTGCTGCGCTGGAGTGACGGCAGTTATCTGGAAGATCAGGATATGT<br>GGCGGATGAGCGGCATTTTCCGTGACGTCTCGTTGCTGCATAAACCGACTACACAAATC<br>AGCGATTTCCATGTTGCCACTCGCTTTAATGATGATTTCAGCCGCGCTGTACTGGAGGCT<br>GAAGTTCAGATGTGCGGCGAGTTGCGTGACTACCTACGGGTAACAGTTTCTTTATGGCA<br>GGGTGAAACGCAGGTCGCCAGCGGCACCGCGCCTTTCGGCGGTGAAATTATCGATGAG<br>CGTGGTGGTTATGCCGATCGCGTCACACTACGTCTGAACGTCGAAAACCCGAAACTGT<br>GGAGCGCCGAAATCCCGAATCTCTATCGTGCGGTGGTTGAACTGCACACCGCCGACGG<br>CACGCTGATTGAAGCAGAAGCCTGCGATGTCGGTTTCCGCGAGGTGCGGATTGAAAAT<br>GGTCTGCTGCTGCTGAACGGCAAGCCGTTGCTGATTCGAGGCGTTAACCGTCACGAGC<br>ATCATCCTCTGCATGGTCAGGTCATGGATGAGCAGACGATGGTGCAGGATATCCTGCTG<br>ATGAAGCAGAACAACTTTAACGCCGTGCGCTGTTCGCATTATCCGAACCATCCGCTGTG<br>GTACACGCTGTGCGACCGCTACGGCCTGTATGTGGTGGATGAAGCCAATATTGAAACCC

	ACGGCATGGTGCCAATGAATCGTCTGACCGATGATCCGCGCTGGCTACCGGCGATGAGC<br>GAACGCGTAACGCGAATGGTGCAGCGCGATCGTAATCACCCGAGTGTGATCATCTGGTC<br>GCTGGGGAATGAATCAGGCCACGGCGCTAATCACGACGCGCTGTATCGCTGGATCAAAT<br>CTGTCGATCCTTCCCGCCCGGTGCAGTATGAAGGCGGCGGAGCCGACACCACGGCCAC<br>CGATATTATTTGCCCGATGTACGCGCGCGTGGATGAAGACCAGCCCTTCCCGGCTGTGC<br>CGAAATGGTCCATCAAAAAATGGCTTTCGCTACCTGGAGAGACGCGCCCGCTGATCCTT<br>TGCGAATACGCCCACGCGATGGGTAACAGTCTTGGCGGTTTCGCTAAATACTGGCAGGC<br>GTTTCGTCAGTATCCCCGTTTACAGGGCGGCTTCGTCTGGGACTGGGTGGATCAGTCGC<br>TGATTAAATATGATGAAAACGGCAACCCGTGGTCGGCTTACGGCGGTGATTTTGGCGAT<br>ACGCCGAACGATCGCCAGTTCTGTATGAACGGTCTGGTCTTTGCCGACCGCACGCCGC<br>ATCCAGCGCTGACGGAAGCAAAACACCAGCAGCAGTTTTTCCAGTTCCGTTTATCCGG<br>GCAAACCATCGAAGTGACCAGCGAATACCTGTTCCGTCATAGCGATAACGAGCTCCTGC<br>ACTGGATGGTGGCGCTGGATGGTAAGCCGCTGGCAAGCGGTGAAGTGCCTCTGGATGT<br>CGCTCCACAAGGTAAACAGTTGATTGAACTGCCTGAACTACCGCAGCCGGAGAGCGCC<br>GGGCAACTCTGGCTCACAGTACGCGTAGTGCAACCGAACGCGACCGCATGGTCAGAA<br>GCCGGGCACATCAGCGCCTGGCAGCAGTGGCGTCTGGCGGAAAACCTCAGTGTGACG<br>CTCCCCGCCGCGTCCCACGCCATCCCGCATCTGACCACCAGCGAAATGGATTTTTGCAT<br>CGAGCTGGGTAATAAGCGTTGGCAATTTAACCGCCAGTCAGGCTTTCTTTCACAGATGT<br>GGATTGGCGATAAAAAACAACTGCTGACGCCGCTGCGCGATCAGTTCACCCGTGCACC<br>GCTGGATAACGACATTGGCGTAAGTGAAGCGACCCGCATTGACCCTAACGCCTGGGTC<br>GAACGCTGGAAGGCGGCGGGCCATTACCAGGCCGAAGCAGCGTTGTTGCAGTGCACG<br>GCAGATACACTTGCTGATGCGGTGCTGATTACGACCGCTCACGCGTGGCAGCATCAGG<br>GGAAAACCTTATTTATCAGCCGGAAAACCTACCGGATTGATGGTAGTGGTCAAATGGCG<br>ATTACCGTTGATGTTGAAGTGGCGAGCGATACACCGCATCCGGCGCGGATTGGCCTGAA<br>CTGCCAGCTGGCGCAGGTAGCAGAGCGGGTAAACTGGCTCGGATTAGGGCCGCAAGA<br>AAACTATCCCGACCGCCTTACTGCCGCCTGTTTTGACCGCTGGGATCTGCCATTGTCAG<br>ACATGTATACCCCGTACGTCTTCCCGAGCGAAAACGGTCTGCGCTGCGGGACGCGCGA<br>ATTGAATTATGGCCCACACCAGTGGCGCGGCGACTTCCAGTTCAACATCAGCCGCTACA<br>GTCAACAGCAACTGATGGAAACCAGCCATCGCCATCTGCTGCACGCGGAAGAAGGCA<br>CATGGCTGAATATCGACGGTTTCCATATGGGGATTGGTGGCGACGACTCCTGGAGCCCG<br>TCAGTATCGGCGGAATTCCAGCTGAGCGCCGGTCGCTACCATTACCAGTTGGTCTGGTG<br>TCAAAAATAATAA(*Hpa*I)

Note: The parentheses before and after the gene indicate the gene or restriction endonuclease site directly connected to it; for the promoter, its characteristic sequence is indicated. The start and stop codons of the gene are marked in red.

Table S3. Plasmid constructs used in this study

Plasmid	Sequence differences of different arsenite-regulated promoters.
P_{J109}-P_{arsWT}	: CCTCTGCACTTACACATTCGTTAAGTCATATATGTTT**TTGACT**TATCCGCTTCGAA-GAGA**GACACT**ACCTGCAACAA***GAATTC***
P_{J109}-$P_{arsWT+24ABS}$	: CCTCTGCACTTACACATTCGTTAAGTCATATATGTTT**TTGACT**TATCCGCTTCGAA-GAGA**GACACT**ACCTGCAACAATTAAGTCATATATGTTTTTGACTTTCCGC ***GAATTC***
P_{J109}-$P_{arsWT+27ABS}$	: CCTCTGCACTTACACATTCGTTAAGTCATATATGTTT**TTGACT**TATCCGCTTCGAA-GAGA**GACACT**ACCTGCAACAATCGTTAAGTCATATATGTTTTTGACTTTCCGC***GAATTC***
P_{J109}-$P_{arsWT+30ABS}$	: CCTCTGCACTTACACATTCGTTAAGTCATATATGTTT**TTGACT**TATCCGCTTCGAA-GAGA**GACACT**ACCTGCAACAACATTCGTTAAGTCATATATGTTTTTGACTTTCCGC***GAATTC***
P_{J109}-$P_{arsWT+32ABS}$	: CCTCTGCACTTACACATTCGTTAAGTCATATATGTTT**TTGACT**TATCCGCTTCGAA-GAGA**GACACT**ACCTGCAACAACACATTCGTTAAGTCATATATGTTTTTGACTTTCCGC***GAATTC***
P_{J109}-$P_{arsWT+33ABS}$	: CCTCTGCACTTACACATTCGTTAAGTCATATATGTTT**TTGACT**TATCCGCTTCGAA-GAGA**GACACT**ACCTGCAACAAACACATTCGTTAAGTCATATATGTTTTTGACTTTCCGC ***GAATTC***
P_{J109}-P_{arsO}	: CCTCTGCACTTACACATTCGTTAAGTCATA**TATGTT**TTTGACTTATCCGCTTCGAA-***GAATTC***
P_{J109}-P_{arsOA1}	: CCTCTGCA**TTTACA**CATTCGTTAAGTCATA**TATGTT**TTTGACTTATCCGCTTCGAA-***GAATTC***
P_{J109}-P_{arsOB1}	: CCTCTGC**TTTACA**ACATTCGTTAAGTCATA**TATGTT**TTTGACTTATCCGCTTCGAA-***GAATTC***
P_{J109}-P_{arsOB2}	: CCTCTGC**TTTACC**ACATTCGTTAAGTCATA**TATGTT**TTTGACTTATCCGCTTCGAA-***GAATTC***
P_{J109}-P_{arsOB3}	: CCTCTGC**TTTAAC**ACATTCGTTAAGTCATA**TATGTT**TTTGACTTATCCGCTTCGAA-***GAATTC***
P_{J109}-P_{arsOB4}	: CCTCTGC**TTGTAC**ACATTCGTTAAGTCATA**TATGTT**TTTGACTTATCCGCTTCGAA-***GAATTC***
P_{J109}-P_{arsOB5}	: CCTCTGC**TTTTAC**ACATTCGTTAAGTCATA**TATGTT**TTTGACTTATCCGCTTCGAA-***GAATTC***
P_{J109}-P_{arsOC1}	: CCTCTG**TTTACA**CACATTCGTTAAGTCATA**TATGTT**TTTGACTTATCCGCTTCGAA-***GAATTC***
P_{J109}-P_{arsOC2}	: CCTCTG**TTGACA**CACATTCGTTAAGTCATA**TATGTT**TTTGACTTATCCGCTTCGAA-***GAATTC***
P_{J109}-P_{arsOC3}	: CCTCTG**TTGACT**CACATTCGTTAAGTCATA**TATGTT**TTTGACTTATCCGCTTCGAA-***GAATTC***
P_{J109}-P_{arsOD1}	: CCTCT**TTTACA**ACACATTCGTTAAGTCATA**TATGTT**TTTGACTTATCCGCTTCGAA-***GAATTC***
P_{J109}-P_{arsOE1}	: CCTC**TTTACA**TACACATTCGTTAAGTCATA**TATGTT**TTTGACTTATCCGCTTCGAA-***GAATTC***
P_{J109}-P_{arsOE2}	: CCTC**TTGACA**TACACATTCGTTAAGTCATA**TATGTT**TTTGACTTATCCGCTTCGAA-***GAATTC***

P$_{J109}$-P$_{arsOF1}$	: CCT**TTTACA**TTACACATTCGTTAAGTCATA**TATGTT**TTTGACTTATCCGCTTCGAA-***GAATTC***
P$_{J109}$-P$_{arsOG1}$	: CC**TTTACA**CTTACACATTCGTTAAGTCATA**TATGTT**TTTGACTTATCCGCTTCGAA-***GAATTC***
P$_{J109}$-P$_{arsOH1}$	: C**TTTACA**ACTTACACATTCGTTAAGTCATA**TATGTT**TTTGACTTATCCGCTTCGAA-***GAATTC***
Plasmid	Description
P$_{lacV}$-P$_{arsWT}$	pPROBE-TT carrying P$_{lacV}$-RBS$_{arsR}$-*arsR*-P$_{arsWT}$ in between *Hin*dIII and *Eco*RI sites
P$_{lacV}$-P$_{arsOB4}$	pPROBE-TT carrying P$_{lacV}$-RBS$_{arsR}$-*arsR*-P$_{arsOB4}$ in between *Hin*dIII and *Eco*RI sites
P$_{lacV}$-P$_{arsOC2}$	pPROBE-TT carrying P$_{lacV}$-RBS$_{arsR}$-*arsR*-P$_{arsOC2}$ in between *Hin*dIII and *Eco*RI sites
P$_{lacV}$-P$_{arsWT}$-*lacZ*	pPROBE-TT carrying P$_{lacV}$-RBS$_{arsR}$-*arsR*-P$_{arsWT}$-*lacZ* in between *Hin*dIII and *Hpa*I sites
P$_{lacV}$-P$_{arsOB4}$-*lacZ*	pPROBE-TT carrying P$_{lacV}$-RBS$_{arsR}$-*arsR*-P$_{arsOB4}$-*lacZ* in between *Hin*dIII and *Hpa*I sites
P$_{lacV}$-P$_{arsOC2}$-*lacZ*	pPROBE-TT carrying P$_{lacV}$-RBS$_{arsR}$-*arsR*-P$_{arsOC2}$-*lacZ* in between *Hin*dIII and *Hpa*I sites

Note: P$_{J109}$-P$_{arsXX}$ (XX means a different name) is the abbreviation of p-TT-P$_{J109}$-r30*arsR*-P$_{arsXX}$. The plasmids shown in sequence illustrate that their arsenic-regulated promoter sequences are different. The bolded sequence represents the original −10 and −35 positions of the wild-type promoter, and the bolded and italicized sequence represents the restriction endonuclease site *Eco*RI. The ArsR binding site is shown in underlined blue font, and the potential −10 site and the reconstructed −35 site are shown in bold red.

Table S4. RNAP binding site library contains eighteen −35 sites and thirty-six −10 sites

	**−35 site**		**−10 site**			
1	TTGACA[3]	CTTAGA[4]	TATAAT[3,4]	GATAAC[4]	TACAAT*	ACAATG[5]
2	TAGACA[3]	ATCTGA[6]	TATAGT[3]	GTTAAA[4]	GACTAT*	TAGGAT[5]
3	TTTACA[3,4]	TAGACT[6]	GATAAT[3]	GTTGTA[4]	TAGGCT[5]	GTATGT[7]
4	TTGTGA[3]	TTTATA*	GATACT[3]	ATATTA[6]	TAATAT[5]	TATATA[7]
5	CTGACA[3]	TTTACG*	TAAATT[3]	TACAGT*	TATGAT[5]	TATCGT[7]
6	TTTACC[4]	GCGGTG[5]	TACTGT[3]	TATTAT*	GTATCT[5]	AGGTG[7]
7	ATTACA[4]	TTGATG[5]	TATGTT[3,4]	GATTAT*	GAGGAT[5]	TAGGTT[7]
8	TTAAGA[4]	TTGCAA	AATAAT[4]	TATTGT*	GATGAT[5]	TTTAAT[7]
9	CTCAGA[4]	TTGACG[7]	GACACT[8]	TACTAT*	TAAAAT[5]	TTAGTA[7]

*Promoters/Catalog/Anderson - parts.igem.org

Table S5. Best fits for the characterized responses of the arsenic sensors with diverse promoters

Expression of ArsR	Promoter	Leaky[a]	k[b] (Fluo/OD_{600})	Km[c]/LOD[d] (ppb)	R^2	Fold change at various conc. (ppb)				
						0.29	1.17	9.38	75	300
P_{J109}	P_{arsWT}	387.71	9798.30	17.46/0.18	0.9837	1.12	1.59	9.39	20.24	25.27
	P_{arsOB4}	84.75	6184.69	18.48/0.27	0.9836	1.20	2.28	24.16	58.43	73.21
	P_{arsOC2}	319.66	16133.81	14.23/0.21	0.9899	1.25	2.38	21.12	43.04	50.54
P_{lacV}	P_{arsWT}	221.11	9065.22	21.84/0.21	0.9868	1.08	1.79	13.66	32.67	41.04
	P_{arsOB4}	40.13	5889.15	22.10/0.31	0.9870	1.09	3.04	45.11	116.04	148.11
	P_{arsOC2}	92.39	16942.84	18.40/0.24	0.9884	1.25	3.90	63.80	147.93	183.52

[a] Leaky: Basal expression in the absence of inducers.

[b] *k*: Maximum expression level in 300 ppb arsenic.

[c] *Km*: Inducer concentration achieving half-maximal activation.

[d] LOD: LOD stands for limit of detection and is the lowest analyte concentration that can probably be reliably distinguished from the basal signal.

Table S6. Accuracy and reliability of the constructed biosensors for real samples

Sample	AFS (ppb)	Final As (ppb)	255 – R[a] （Dilution factor）	Estimated (ppb)	Recover
G-Sample 1	20.7	20.7	129 (6)	17.82	86.08%
G-Sample 2		50.7	148 (10)	45.88	90.49%
G-Sample 2		80.7	137.33 (20)	76.07	94.26%

[a]Mean 255 – R value (n = 3).

Table S7. Comparison of previously reported arsenic sensors with this study

**Microbial chassis Biosensor**	**LOD (ppb)[a]**	**Linear or input range (ppb)[b]**	**Description，reference**
***gfp* reporter**			
E.coli DH5α P_{lacV}-P_{arsOC2}	0.24	2.34-150	This study
E.coli DH5α pPR-arsR-ABS	7.5	7.5-75	Gray value analysis, *ars* operon from R773 plasmid of *E. coli*.[9]
E.coli DH5α pVLAS1	7.5	37.5-450	Non-specificity, high background noise, *ars* operon from pI258 plasmid of *S. aureus*.[10]
E.coli DH5α pUC18-ep3ars-gfp	0.75	1-50	Flow cytometry analysis, directed evolution *ars* operon, *ars* operon from pPR-arsR-ABS.[11]
E. coli K-12	5	5-140	With a T7 RNAP amplifier, ars operon from *E.*

POLA			*coli* genome.[12]
E. coli TOP10 pAsS	0.75	2.3-7.5	Low fold changes, narrow response range. *ars* operon from *G. sulfurreducens* genome.[13]
Cell-free ep3-*gfp*	3.65	4-50	Cell-free expression, fast response, *ars* operon from pUC18-ep3ars-gfp.[14]
***lacZ* reporter**			
E.coli DH5α P_{lacV}-P_{arsOC2}-*lacZ*	0.39	1-5	This study
E.coli pMV-arsR-ABS	8	8-78	Semi-quantitative analysis, *ars* operon from R773 plasmid of *E. coli*.[9]
E. coli JM109 Pars/arsR-lacZ	5	5-20	Output pH change, *ars* operon from *E. coli* genome.[15]
B. subtilis ars-23	7.5	7.5-7500	Coupled enzyme reaction for luminescence, *ars* operon from *B. subtilis*.[16]
E. coli DH5α strain 2245	0.8	3.75-30	Electrochemical read-out, contain pPR-arsR-ABS-RBS-lacZ, with RBS optimal.[17]
E.coli DH5α pAs-lacZ	10	10-500	High background, *E. coli* DH5α, *ars* operon from pI258 plasmid of *S. aureus*.[18]

[a] The LOD indicated in the paper, or the lowest concentration actually used.

[b] The linear range concentration indicated in the paper, or the actual concentration range used.

References

1. Weel-Sneve, R.; Kristiansen, K. I.; Odsbu, I.; Dalhus, B.; Booth, J.; Rognes, T.; Skarstad, K.; Bjoras, M., Single transmembrane peptide DinQ modulates membrane-dependent activities. *PLoS Genet* **2013,** *9* (2), e1003260.

2. Chun Xu, W. S., and Barry P. Rosen, The Chromosomal arsR Gene of Escherichia coli Encodes a trans-acting Metalloregulatory Protein. *J Biol Chem* **1996,** *271* (5), 2427–2432.

3. Chen, Y.; Ho, J. M. L.; Shis, D. L.; Gupta, C.; Long, J.; Wagner, D. S.; Ott, W.; Josic, K.; Bennett, M. R., Tuning the dynamic range of bacterial promoters regulated by ligand-inducible transcription factors. *Nat Commun* **2018,** *9* (1), 64.

4. Brewster, R. C.; Jones, D. L.; Phillips, R., Tuning promoter strength through RNA polymerase binding site design in Escherichia coli. *PLoS Comput Biol* **2012,** *8* (12), e1002811.

5. Davis, J. H.; Rubin, A. J.; Sauer, R. T., Design, construction and characterization of a set of insulated bacterial promoters. *Nucleic Acids Res* **2011,** *39* (3), 1131-41.

6. Cox, R. S., 3rd; Surette, M. G.; Elowitz, M. B., Programming gene expression with combinatorial promoters. *Mol Syst Biol* **2007,** *3*, 145.

7. Stanton, B. C.; Nielsen, A. A.; Tamsir, A.; Clancy, K.; Peterson, T.; Voigt, C. A., Genomic mining of prokaryotic repressors for orthogonal logic gates. *Nat Chem Biol* **2014,** *10* (2), 99-105.

8. Chen, S. Y.; Wei, W.; Yin, B. C.; Tong, Y.; Lu, J.; Ye, B. C., Development of a Highly Sensitive Whole-Cell Biosensor for Arsenite Detection through Engineered Promoter Modifications. *ACS Synth Biol* **2019,** *8* (10), 2295-2302.

9. Stocker, J.; Balluch, D.; Gsell, M.; Harms, H.; Feliciano, J.; Da Unert, S.; Malik, K. A.; Roelof, Development of a set of simple bacterial biosensors for quantitative and rapid measurements of arsenite and arsenate in potable water.

Environ Sci Technol **2003,** *37* (20), 4743-4750.

10. Liao, V. H.; Ou, K., Development and testing of a green fluorescent protein-based bacterial biosensor for measuring bioavailable arsenic in contaminated groundwater samples. *Environ Toxicol Chem* **2005,** *24*.

11. Li, L.; Liang, J.; Hong, W.; Zhao, Y.; Sun, S.; Yang, X.; Xu, A.; Hang, H.; Wu, L.; Chen, S., Evolved bacterial biosensor for arsenite detection in environmental water. *Environ Sci Technol* **2015,** *49* (10), 6149-55.

12. Pola-López, L. A.; Camas-Anzueto, J. L.; Martínez-Antonio, A.; Luján-Hidalgo, M. C.; Anzueto-Sánchez, G.; Ruíz-Valdiviezo, V. M.; Grajales-Coutiño, R.; González, J. H. C., Novel arsenic biosensor "POLA" obtained by a genetically modified E. coli bioreporter cell. *Sensor Actuat B-Chem* **2018,** *254*, 1061-1068.

13. Li, P.; Wang, Y.; Yuan, X.; Liu, X.; Liu, C.; Fu, X.; Sun, D.; Dang, Y.; Holmes, D. E., Development of a whole-cell biosensor based on an ArsR-P regulatory circuit from Geobacter sulfurreducens. *Environ Sci and Ecotechnol* **2021,** *6*.

14. Wang, X.; Zhu, K.; Chen, D.; Wang, J.; Wang, X.; Xu, A.; Wu, L.; Li, L.; Chen, S., Monitoring arsenic using genetically encoded biosensors in vitro: The role of evolved regulatory genes. *Ecotoxicol Environ Saf* **2021,** *207*, 111273.

15. Aleksic, J.; de Mora, K.; Millar, A.; Davidson, B.; Kozma-Bognar, L.; Ma, H.; French, C.; Bizzari, F.; Elfick, A.; Wilson, J.; Cai, Y.; Seshasayee, S. L.; Nicholson, J.; Ivakhno, S., Development of a novel biosensor for the detection of arsenic in drinking water. *IET Synthetic Biology* **2007,** *1* (1), 87-90.

16. Date, A.; Pasini, P.; Sangal, A.; Daunert, S., Packaging Sensing Cells in Spores for Long-Term Preservation of Sensors: A Tool for Biomedical and Environmental Analysis. *Anal Chem* **2010,** *82* (14), 6098-6103.

17. Cortes-Salazar, F.; Beggah, S.; van der Meer, J. R.; Girault, H. H., Electrochemical As(III) whole-cell based biochip sensor. *Biosens Bioelectron* **2013,** *47*, 237-42.

18. Huang, C. W.; Wei, C. C.; Liao, V. H., A low cost color-based bacterial biosensor for measuring arsenic in groundwater. *Chemosphere* **2015,** *141*, 44-9.
